# Supplementary material for: Expiratory Technique versus Tracheal Suction to Obtain Good-Quality Sputum from Patients with Suspected Lower Respiratory Tract Infection: A Randomized Controlled Trial
Source: Diagnostics (Basel). 2022 Oct 16;12(10):2504. doi: 10.3390/diagnostics12102504 (PMC9600387; doi:10.3390/diagnostics12102504)
Supplement: Supplementary file 1 [file diagnostics-12-02504-s001.zip › diagnostics-1973601-supplementary.pdf]

# Expiratory technique versus tracheal suction to obtain good-quality sputum from patients with suspected lower respiratory tract infection: a randomized controlled trial

Supplementary Material - Sensitivity and sub-analyses

## Table of contents


**I) Intention to treat analysis** ..... 1

**II) Complete case analysis** ..... 1

**III) Sensitivity analysis of adverse events** ..... 2

**IV) Harms (text S4)** ..... 2

**V) Sensitivity analysis of the patient experience of the sputum collection procedure** ..... 4

**VI) Descriptive analysis from tracheal suctions obtained from patients unable to expectorate** ..... 6


## I) Intention to treat analysis

| Outcome | Unadjusted | p-value | Adjusted | p-value |
|---|---|---|---|---|
| **Sputum quality** | OR 1.83 (95%CI 1.05 to 3.19) | 0 .035 | OR 1.45 (95% CI 0.78 to 2·70) | 0.233 |

*Table S1: Intention to treat analysis unadjusted and adjusted for antibiotics, pneumonia, smoking, SARS-CoV-2 (Severe Acute Respiratory Syndrome Coronavirus 2), CURB-65 ( Confusion, Urea, Respiratory rate, Blood pressure and age >65), and age.*

## II) Complete case analysis

| Outcome | Unadjusted | p-value | Adjusted | p-value |
|---|---|---|---|---|
| **Sputum quality** | OR 2.42 (95%CI 1.31 to 4.47) | 0.005 | OR 1.96 (95%CI 0.98 to 3.90) | 0.055 |
| **Adverse effects** | IRR 1.02 (95%CI 0.87 to 1.19) | 0.796 | IRR 0.99 (95%CI 0.79 to 1.25) | 0.985 |
| **Patient experience** | N/A | < 0·001 | N/A | < 0.001 |

*Table S2: Complete case analysis unadjusted and adjusted for antibiotics, pneumonia, smoking, SARS-CoV-2 (Severe Acute Respiratory Syndrome Coronavirus 2), CURB-65 ( Confusion, Urea, Respiratory rate, Blood pressure and age >65), and age.*

## III) Sensitivity analysis of adverse events

| Adverse Events | TS | | FETIS | | p-value |
|---|---|---|---|---|---|
| | Total, n | Event, n (%) | Total, n | Event, n (%) | |
| **Vital parameters** | | | | | |
| Oxygen saturation* | 141 | 21 (15%) | 139 | 26 (19%) | 0.426 |
| Respiratory rate** | 141 | 5 (4%) | 139 | 3 (2%) | 0.722 |
| **Side effects** | | | | | |
| Procedure related bleeding | 141 | 16 (11%) | 139 | 1 (0·7%) | 0 .0002† |
| Bronchospasm | 141 | 3 (2%) | 139 | 2 (1%) | 1.000 |
| Others*** | 141 | 5 (4%) | 139 | 2 (1%) | 0 .447 |
| **Patients symptoms** | | | | | |
| Cough | 141 | 6 (4%) | 139 | 7 (5%) | 0.784 |
| Dyspnea | 141 | 18 (13%) | 139 | 7 (5%) | 0 .034† |
| Chest tightness | 141 | 3 (2%) | 139 | 6 (4%) | 0.333 |
| Sputum | 141 | 6 (4%) | 139 | 9 (7%) | 0 .439 |
| **CR 10 Borg scale** | | | | | |
| CR10 report | 141 | 22 (16%) | 139 | 16 (12%) | 0 .383 |
| **Mortality** | 138 | 2 (1%) | 139 | 4 (3%) | 0 .684 |
| **Readmission** | 134 | 40 (28%) | 134 | 34 (25%) | 0 .494 |

*Table S3: Sensitivity analysis of each adverse event included in pooled adverse event analysis. Number=n and percentage (%).*
Fishers Exact and $\chi^2$ test was used to compare the adverse event variables.
Adverse events were reported during and at the latest 10 min after the procedure.
Aggravation of vital parameters: *Oxygen saturation decreased to ≤ 93% (Chronic Obstructive Pulmonary Disease patients ≤ 88%), **Respiratory rate decreased to ≤ 12 or increased to higher than 20 times per minute. Patient reported aggravation of symptoms measured by each symptom (cough, dyspnea, chest tightness and sputum) and measured by Borg scale CR10. Mortality was measured within 7 days from admission and readmission within one month from discharge.
***Others adverse events reported were nausea and vomiting.
† p-value significant < .05

## IV) Harms (text S1)

After the analyses, the principal investigator reviewed all medical records for patients that experienced procedure-related bleedings, dyspnea, bronchospasms, decrease in oxygen saturation, and worsening of respiratory rate. This review ensured agreement between the project data and descriptions in the patient's medical record. An infectious disease and emergency medicine expert was consulted if there were discrepancies. Even though there was a difference between groups regarding bleedings and dyspnea, these adverse events were reported as mild, short-lived without the need for physician consultation except for one case described in detail below. In addition,

worsening related oxygen saturation and respiratory rate were short-lived in patients treated with oxygen treatment and delivered a sample without oxygen.

Aggravation of dyspnea, decrease in oxygen saturation and bleeding:
One critically ill patient who was allocated the tracheal suction group experienced several symptoms at once (bleeding, dyspnea, oxygen desaturation) and consultation with a senior physician was required. The specialist in infectious disease and emergency medicine expert reviewed this medical record and assessed the patient's condition as critical from arrival. The worsening of the patient's vital parameters was not deemed a consequence of the tracheal suction procedure.

Bleeding:
One patient allocated to the intervention group and was unable to produce expectorate. This patient underwent tracheal suction and experienced bleeding from the nose and mouth in moderate severity and needed supervision until the condition was stabilized.

Bronchospasm:
Two patients experienced mild bronchospasms possibly due to treatment with b2-agonist prior to saline inhalation. One patient had no comorbidity and the other was a patient with chronic respiratory disease and a history of asthma. One patient in the intervention group required additional treatment 10 minutes after sputum induction due to a more severe bronchospasm.

## V) Sensitivity analysis of the patient experience of the sputum collection procedure

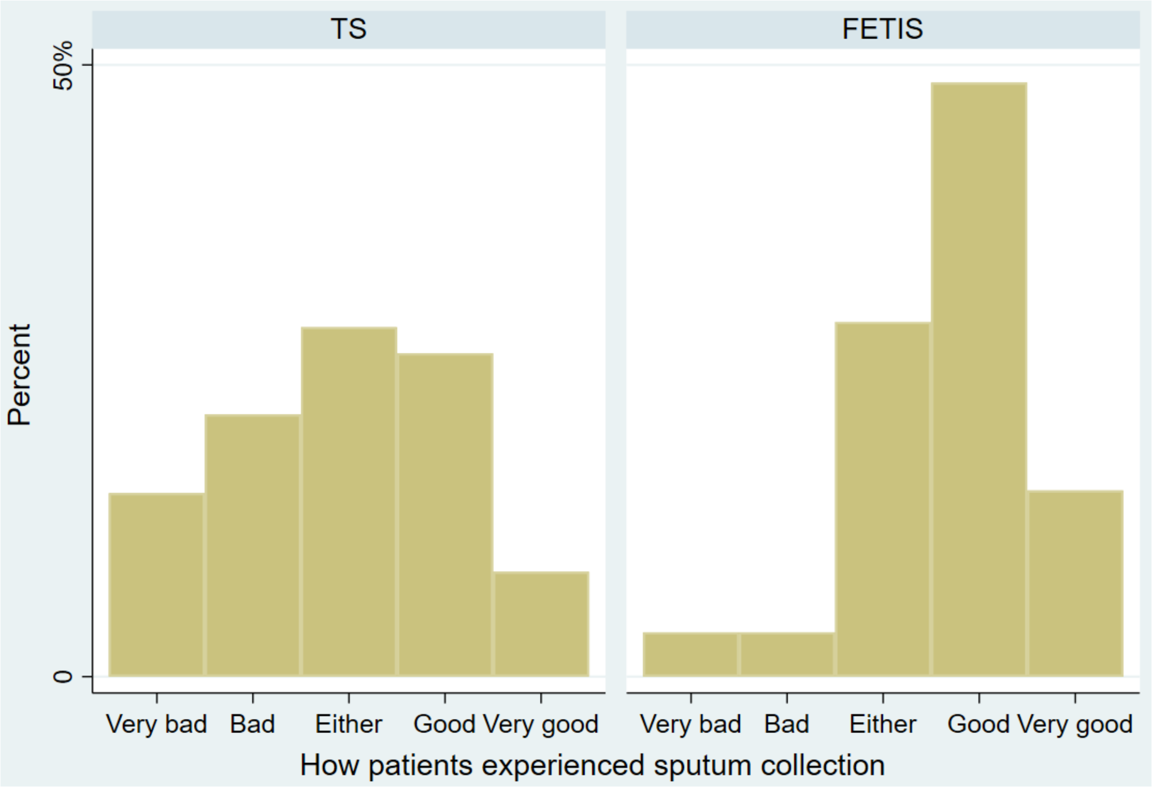


*Figure S1: Distribution from Likert scale of patients experience. Comparison between the two groups of how patients experienced sputum collection p< 0.001 whereby 140 (99%) patients in the TS group and 138 (99%) from the intervention group answered the question.*

Even though there were significant differences between groups p<0.0001, there was only a slight difference between groups of the neutral answer where the mean difference between groups was less than one 2.9 for TS and 3.6 for FETIS group.

| Collecting method | TS (102) | FETIS (82) |
|---|---|---|
| **Likert scale** | | |
| Very bad | 21 (81%) | 5(19%) |
| Bad | 30 (86%) | 5 (14%) |
| Either bad or good | 40 (50%) | 40 (50%) |
| Good | 37 (36%) | 67 (64%) |
| Very good | 12 (36%) | 21 (64%) |
| **Explanations*** | 91 (55%) | 74 (45%) |
| Painful and unpleasant | 20 (22%) | 4 (5%) |
| Breathless | 6 (7%) | 0 (0%) |
| Ineffective | 2 (2%) | 13 (18%) |
| Quickly | 25 (27%) | 2 (3%) |
| Acceptable | 17 (19%) | 21 (28%) |
| Easier breathing | 5 (5%) | 10 (13%) |
| Facilitate expectoration | 0 (0%) | 7 (9%) |
| Good information and a professional approach | 10 (11%) | 7 (9%) |
| Important for further treatment | 6 (7%) | 1 (2%) |
| Enjoyable | 0 (0%) | 9 (12%) |

*Table S4: Patient's explanation of their choice from Likert scale*
*Patients can contribute with more than one explanation based on Likert scale

## VI) Descriptive analysis from tracheal suctions obtained from patients unable to expectorate

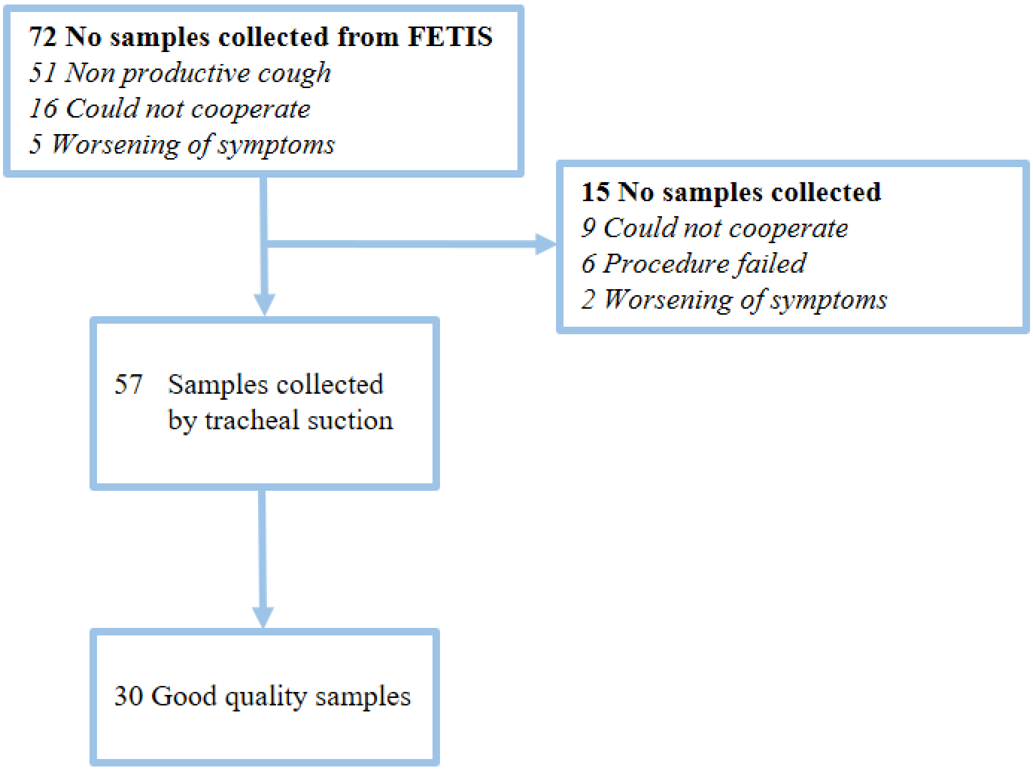


*Figure S2: Profile of the population with no expectorated samples*

| Adverse events | Event, n (%) |
|---|---|
| **Procedure-related bleedings** | 4 (7%) |
| **Bronchospasms (interrupted procedure)** | 4 (7%) |
| **Aggravation of dyspnea** | 2 (3%) |

*Table S5: Harms reported from 57 TS from patients that were unable to expectorate from FETIS group*

As described in the harm section above, one critically ill patient from FETIS group unable to expectorate experienced several adverse events after a tracheal suction procedure (bleeding, dyspnea, oxygen desaturation) where it was necessary to consult a senior physician. All others reported adverse events were considered as mild without the need for further treatment.
